# Pseudoaneurysm associated with arteriovenous fistula involving a superficial breast arteriole after vacuum-assisted removal of a benign mass: A case report

**DOI:** 10.1097/MD.0000000000012250

**Published:** 2018-09-07

**Authors:** Yixuan Li, Zhoupeng Wu, Feng Yan, Yulan Peng, Lang Ma, Guojun Zeng, Qing Lv

**Affiliations:** a Department of Ultrasound; b Department of Vascular Surgery; c Department of Laboratory of Clinical Ultrasound Imaging Drug Research; d Department of Breast and Thyroid Surgery, West China Hospital of Sichuan University, Chengdu, Sichuan, China.

**Keywords:** arteriovenous fistula, endovascular therapy, percutaneous embolization, pseudoaneurysm, surgery

## Abstract

**Rationale:**

Pseudoaneurysm (PA) with associated arteriovenous fistula (AVF) is most often secondary to vascular catheterization, percutaneous biopsy, surgery, or trauma. PA-AVF occurs mainly in large or median arterial territories but rarely in the superficial arterioles of the breast.

**Patient concerns:**

A 30-year-old woman underwent vacuum-assisted removal of breast fibroadenomas under ultrasonic guidance. On the follow-up visit, the patient complained of a painful enlarging lump in her left breast.

**Diagnoses:**

An iatrogenic breast PA-AVF was diagnosed.

**Interventions:**

The patient was treated with surgical excision and ligation under local anesthesia.

**Outcomes:**

At the 1-month follow-up, the wound was found to have healed well, and breast PA-AVF was eradicated.

**Lessons:**

Vacuum-assisted removal has been the first-line intervention for benign mass resection because of minimal invasion, but the risk of serious vascular complications remains. Careful duplex ultrasound examination prior to the procedure is highly recommended.

## 1 Introduction

Pseudoaneurysm with associated arteriovenous fistula (PA-AVF) occurs mainly in large or medium-sized head-and-neck arteries, lower extremity arteries, and visceral arteries. PA-AVF of a superficial breast arteriole is rare. Ultrasound-guided vacuum-assisted removal (VAR) is a minimally invasive technique for breast benign masses that is now extensively used. Reported complications of VAR are infrequent, with hematoma and infection figuring as the most common complications.^[1]^ A vascular complication, particularly PA-AVF of a superficial breast arteriole, is exceedingly rare but must be considered to ensure appropriate treatment. The published literature only identified 1 case of PA-AVF of a superficial breast arteriole that was occluded with percutaneous coiling under angiography.^[2]^ We determined that treatment options for breast PA-AVF involve 3 factors, that is, the interval between initial injury and detection, lesion size, and the distance between the AVF and associated PA. We describe a case of large PA-AVF following VAR of breast fibroadenomas that was treated with surgical excision and ligation. Written informed consent was obtained from the patient. This case report was approved by the West China Hospital of Sichuan University Research Ethics Board.

## 2 Case report

A 30-year-old woman without significant history underwent ultrasonography, which revealed 2 benign-appearing masses in the left breast. VAR of the masses was performed using an 8-G needle under ultrasonic guidance. Histology confirmed fibroadenomas.

**Figure 1:**
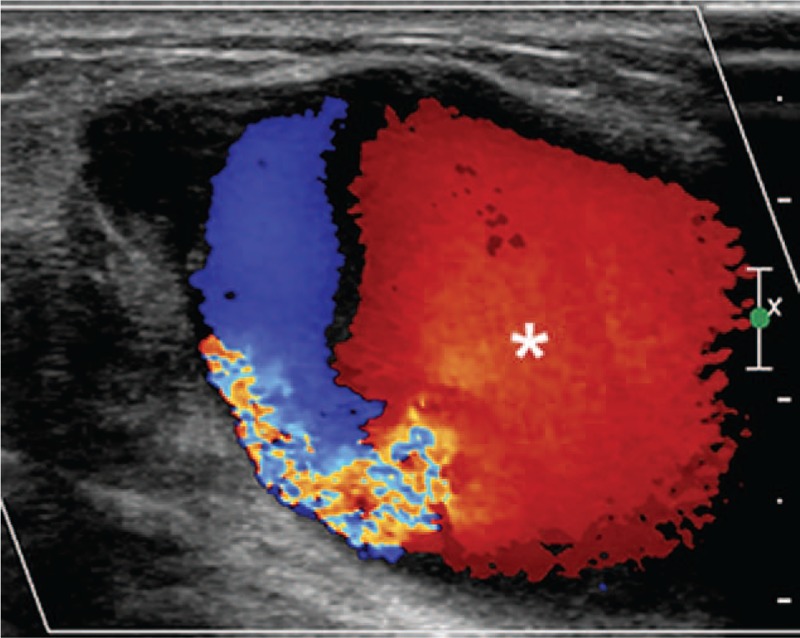
Color Doppler imaging revealed an anechoic mass with typical whirling blood flow and a red and blue, biphasic “to-and-fro” pattern, suggesting pseudoaneurysm (asterisk).

**Figure 2:**
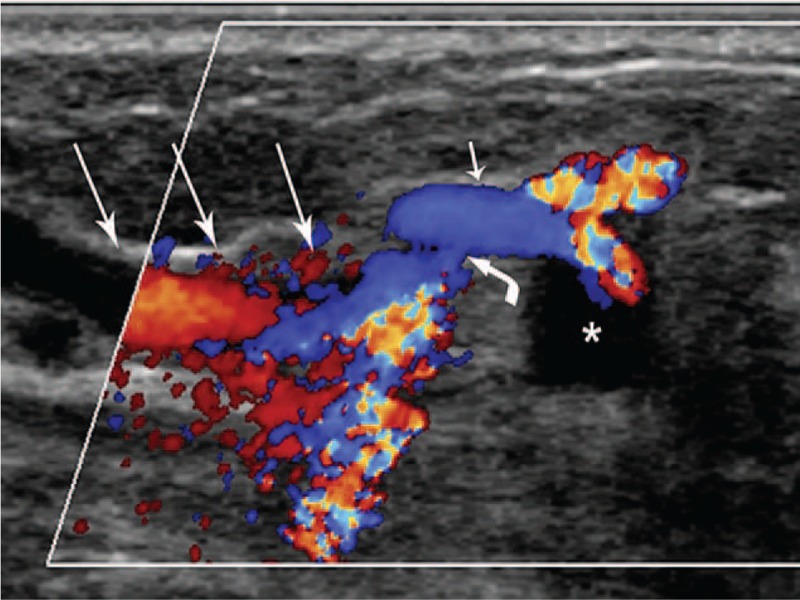
Another color Doppler imaging section showed the pseudoaneurysm (asterisk) with associated arteriovenous fistula, the artery feeding the pseudoaneurysm (short arrow), the concomitant vein (long arrow), and the fistula (bent arrow) communicating with the feeding artery and concomitant vein, suggesting pseudoaneurysm with associated arteriovenous fistula.

Three months later, the patient returned for a follow-up visit, complaining of a painful, enlarging lump in her left breast. Clinical examination revealed a pulsatile mass with a continuous machinery murmur in the outer upper quadrant of the left breast. Color Doppler imaging demonstrated an anechoic lump (45 × 26 × 33 mm) fed by an afferent artery; the lump showed a yin-yang appearance and biphasic “to-and-fro” pattern (Fig. 1). A fistula was situated 3-mm proximal to the anechoic lump, connecting the afferent artery and a concomitant vein (Fig. 2). Based on these findings, the patient was diagnosed with breast PA-AVF.

Multidisciplinary consultation concluded that the lesion posed a risk of rupture and should be excised and ligated under local anesthesia as soon as possible. A 3-mm operative incision was made along the direction of the vascular route under ultrasonography guidance. The incision was at 1 o’clock position, 7-cm from the nipple, and was concealed to prevent a cosmetic defect. The proximal and distal artery and vein of the PA-AVF were ligated. The PA-AVF was excised. The entire operation was very simple and time-efficient. The mean bleeding volume was only 5 mL. At the 1-month follow-up, the wound was found to have healed well, and ultrasonography confirmed disappearance of the breast PA-AVF.

## 3 Discussion

The most common complications of VAR include hematoma and infection. Vascular injuries, particularly PA and AVF formation, are infrequent. The incidence of PA is estimated to occur in 0.05% to 0.5% of all diagnostic and therapeutic puncture operations.^[3]^ The reported incidence rates of newly diagnosed AVF have varied in different studies from 0.89 to 1.34 cases per 100,000 person-years.^[4]^ PA-AVF of a superficial breast arteriole is exceedingly rare, without reported incidence. We only found 1 case of breast PA-AVF in the literature.

We speculated that the breast PA-AVF in our patient was caused by angular penetration of the 8-G needle into the bilateral arterial wall and one side of the concomitant vein during vacuum-assisted removal of the fibroadenomas. At one side of the perforated arterial wall, blood exuded into adjacent tissues, and subsequently formed a fibrous encapsulation, which ultimately developed into the PA connecting with the arterial lumen. The AVF developed at the other side of the perforated arterial and venous wall. The AVF was situated 3 mm proximal to the PA.

Management options for PA-AVF generally include endovascular covered stenting, percutaneous thrombin or alcohol injection, endovascular/percutaneous coil embolization, sonographically guided compression, and surgical repair.^[5]^ In our case, we excluded the endovascular procedures because of the fine and tortuous superficial arterioles of the breast, which could make it technically difficult. Next, because of the close distance between PA and AVF, we excluded percutaneous thrombin or alcohol injection, which had a risk of inducing vascular or pulmonary embolism via the fistula.^[6]^ Lee et al successfully performed percutaneous coiling under angiography to occlude a superficial small PA-AVF of the breast.^[2]^ Hadzimehmedagic et al used catheterization and coiling to embolize a 40-mm PA-AVF of the deep femoral artery and vein.^[7]^ However, the coil became displace and conventional surgery was finally performed. In our case, the large size of the PA-AVF and good mobility of the breast increased the risk of transposition and recurrence after percutaneous microcoiling. In addition, percutaneous embolization takes longer than surgical repair for mass reduction.^[8]^ Therefore, we chose surgical repair under local anesthesia, which proved simple and effective.

Our review of the literature suggests that the treatment options for breast PA-AVF depend on the interval between initial injury and detection, lesion size, and the distance between AVF and PA. Stable, small, and asymptomatic breast PA-AVF measuring <20 mm can thrombose spontaneously within 3 months after initial injury, and can be managed with conservative observation or compression therapy.^[5,9]^ Breast PA-AVF measuring <30 mm and persisting after 3 months may be treated effectively with percutaneous microcoiling.^[10]^ A breast PA-AVF measuring >30 mm may have higher risk of recurrence and may be treated more effectively using surgery under local anesthesia.^[1,11]^

## 4 Conclusion

In conclusion, careful duplex ultrasound examination prior to VAR is highly recommended to avoid unwanted vascular injuries. When breast PA-AVF develops, we should consider the interval between initial injury and detection, lesion size, and the distance between AVF and PA to choose optimal treatment.

## Author contributions

**Conceptualization:** Yixuan Li, Yulan Peng.

**Data curation:** Yixuan Li, Guojun Zeng, Zhoupeng Wu.

**Formal analysis:** Feng Yan.

**Funding acquisition:** Yulan Peng.

**Investigation:** Guojun Zeng, Lang Ma.

**Resources:** Qing Lv, Yulan Peng.

**Software:** Feng Yan, Lang Ma.

**Supervision:** Yulan Peng, Qing Lv.

**Validation:** Yulan Peng.

**Visualization:** Feng Yan.

**Writing – original draft:** Yixuan Li.

**Writing – review & editing:** Yixuan Li, Zhoupeng Wu, Zhoupeng Wu, Feng Yan, Yulan Peng.

Author name: 0000-0003-3077-0746.
